# Low-Dose Bevacizumab for the Treatment of Focal Radiation Necrosis of the Brain (fRNB): A Single-Center Case Series

**DOI:** 10.3390/cancers15092560

**Published:** 2023-04-29

**Authors:** Jens Tijtgat, Evan Calliauw, Iris Dirven, Manon Vounckx, Randa Kamel, Anne Marie Vanbinst, Hendrik Everaert, Laura Seynaeve, Dirk Van Den Berge, Johnny Duerinck, Bart Neyns

**Affiliations:** 1 Department of Medical Oncology, UZ Brussel, Laarbeeklaan 101, 1090 Brussels, Belgium; 2 Department of Radiotherapy, UZ Brussel, Laarbeeklaan 101, 1090 Brussels, Belgium; 3 Department of Medical Imaging, UZ Brussel, Laarbeeklaan 101, 1090 Brussels, Belgium; 4 Department of Nuclear Medicine, UZ Brussel, Laarbeeklaan 101, 1090 Brussels, Belgium; 5 Department of Neurology, UZ Brussel, Laarbeeklaan 101, 1090 Brussels, Belgium; 6 Department of Neurosurgery, UZ Brussel, Laarbeeklaan 101, 1090 Brussels, Belgium

**Keywords:** radiation necrosis of the brain, bevacizumab, low-dose regimen, stereotactic radiation therapy, radiotherapy, case series, VEGF inhibitor

## Abstract

**Simple Summary:**

Focal radiation necrosis of the brain (fRNB) is a late side effect that can occur after treatment of brain lesions with focal radiation therapy (stereotactic radiosurgery [SRS] or stereotactic radiation therapy [SRT]). This is becoming more common as more cancer patients are receiving effective systemic therapy for brain metastases, extending survival, and putting them at risk for fRNB. Currently, treatment options are limited to long-term corticosteroid therapy, which has significant side effects, or surgery with its inherent risks. Bevacizumab, a monoclonal antibody that targets the vascular endothelial growth (VEGF), is effective in treating fRNB but its use has remained limited due to its cost. In this single-center case series, a fixed low dose of bevacizumab (400 mg loading dose followed by 100 mg every 4 weeks) was shown to be a safe and cost-effective alternative treatment option for fRNB.

**Abstract:**

Focal radiation necrosis of the brain (fRNB) is a late adverse event that can occur following the treatment of benign or malignant brain lesions with stereotactic radiation therapy (SRT) or stereotactic radiosurgery (SRS). Recent studies have shown that the incidence of fRNB is higher in cancer patients who received immune checkpoint inhibitors. The use of bevacizumab (BEV), a monoclonal antibody that targets the vascular endothelial growth factor (VEGF), is an effective treatment for fRNB when given at a dose of 5–7.5 mg/kg every two weeks. In this single-center retrospective case series, we investigated the effectiveness of a low-dose regimen of BEV (400 mg loading dose followed by 100 mg every 4 weeks) in patients diagnosed with fRNB. A total of 13 patients were included in the study; twelve of them experienced improvement in their existing clinical symptoms, and all patients had a decrease in the volume of edema on MRI scans. No clinically significant treatment-related adverse effects were observed. Our preliminary findings suggest that this fixed low-dose regimen of BEV can be a well-tolerated and cost-effective alternative treatment option for patients diagnosed with fRNB, and it is deserving of further investigation.

## 1. Introduction

Stereotactic radiosurgery (SRS) and stereotactic radiotherapy (SRT) are increasingly used in the treatment of both benign and malignant (primary or metastatic) brain lesions. SRS and SRT irradiate brain lesions more accurately while sparing healthy brain structures compared to whole-brain radiation therapy (WBRT). Nevertheless, because radiation dosing delivered to the tumor and surrounding normal brain can be higher using these methods, both SRS and SRT have a potential important side effect under the form of focal radiation necrosis of the brain (fRNB) [1,2]. The incidence of fRNB is estimated to be between 7–24% [3,4,5,6]. Signs and symptoms include neurological deficits, cognitive decline, increased intracranial pressure and/or seizures [7]. The primary pathogenesis of fRNB is not yet fully understood, but damage to small blood vessels is suspected to be the principal cause [8,9]. Excessive production of vascular endothelial growth factor A (VEGF-A) and the associated neo-angiogenesis cause the formation of edema and worsening of brain tissue hypoxia and necrosis [10,11,12].

The main risk factor associated with the development of fRNB is the cumulative radiation dose, also taking into account re-irradiation [13,14]. Furthermore, fRNB is more often seen after SRS as compared to SRT, indicating a risk-lowering effect of fractionation [15]. It has recently been noted that the incidence of fRNB following SRT/SRS of brain metastases from solid tumors has been increasing in patients who received immune checkpoint inhibitors (ICI, such as anti PD-(L)1 and anti CTLA-4 monoclonal antibodies (mAb)) and possibly also targeted therapies [16,17]. In these cases, fRNB tends to occur after a median interval of 1 year (range: 8 to 46 months) following SRS/SRT [16,17,18,19,20]. Du Four et al. reported an increased incidence (12.8%) of fRNB in a series of 142 advanced melanoma patients treated with the PD-1 inhibiting mAb pembrolizumab after having previously undergone SRS/SRT [16]. In another cohort of 135 melanoma patients, almost 20% of melanoma patients that received SRS/SRT and anti-PD-1 who survived beyond 1 year developed fRNB [21]. Based on another series from our group, fRNB may also be more frequent after treatment with BRAF-/MEK inhibitors in patients treated for BRAF V600-mutant melanoma brain metastases with SRS/SRT.

The differential diagnosis between fRNB and recurring diseases can be challenging. Pathology remains the gold standard for making a diagnosis. Advanced magnetic resonance imaging (MRI), including MR-spectroscopy and perfusion MRI, can be useful for establishing the diagnosis without the need for a surgical intervention [22]. ^18^F-fluoro-deoxy-D-glucose positron emission tomography/computed tomography (^18^F-FDG PET-CT) imaging of the brain can be of additional value, as fRNB will be characterized by decreased uptake of ^18^F-FDG compared to malignant lesions and surrounding healthy brain tissue [22,23,24].

Currently, high doses of corticosteroids are most often used as first-line therapy for symptomatic patients that are not good candidates for neurosurgical debridement. By decreasing the secretion of pro-inflammatory cytokines and reducing the blood–brain barrier permeability, corticosteroids reduce cerebral edema and related symptoms. However, the side effects of prolonged administration of high-dose corticosteroids, which is often necessary to treat fRNB, are common. These include myopathy, hyperglycemia, atrophy of the skin, weight gain, gastritis, and immunosuppression [25,26]. The surgical debridement of fRNB can offer a permanent solution, but its indication is limited to patients that are eligible for a safe neurosurgical intervention in non-eloquent regions of the brain and it inherently carries a risk for the deterioration of neurological symptoms [27,28].

In 2014, Tye et al. reported that the vascular endothelial growth factor A (VEGF-A), blocking monoclonal antibody bevacizumab (BEV) was an effective new therapeutic option for fRNB [29]. BEV was the first approved angiogenesis inhibitor. It is approved at a dose of 5 to 10 mg/kg every 2 weeks as a component of several chemotherapy regimens for advanced solid tumors [30]. When used to treat fRNB, it allows to reduce or replace the need for corticosteroids and avoid the complications of their long-term use [26,29,31]. Despite its well-known potential side effects, such as hypertension and decreased wound healing, BEV is a well-tolerated medical therapy. Several small studies demonstrated that doses between 5 to 7.5 mg/kg, reflecting the approved dosing regimens for oncological indications, reduce neurological symptoms and cerebral edema [32,33].

Bevacizumab has (as of 2022) not yet been registered for the treatment of fRNB, and its high drug cost has been prohibitive in its use in this indication. Gordon et al. made the observation that significantly lower doses of bevacizumab (down to 0.3 mg/kg) than those approved for oncological indications, reduced free serum VEGF concentrations below the detection limit of the assay [34]. Based on these findings, we started using a low-dose bevacizumab regimen (400 mg I.V. loading dose followed by 100 mg I.V. Q4w) for the treatment of patients diagnosed with symptomatic fRNB not eligible for neurosurgical debridement [34]. The initial reports of this regimen were presented at the 2021 ESMO-congress (European Society for Medical Oncology) [35]. Recently, other research groups demonstrated the efficacy of a low dose of bevacizumab, including regimens of 1 mg/kg and 3 mg/kg [36,37]. Here, we report a retrospective analysis of patients receiving treatment with this low-dose bevacizumab regimen at our center between 2016 and 2022.

## 2. Materials and Methods

### 2.1. Patient Selection, Clinical Outcomes, and Adverse Events

We conducted a single-center, retrospective identification of patients with benign or malignant cerebral lesions who received SRT or SRS, subsequently developed symptomatic fRNB and were treated with a low-dose bevacizumab regimen at the UZ Brussel hospital (Belgium) between 2016 and 2022. All patients that received at least one administration of 100 mg bevacizumab I.V were included. Patients were diagnosed with fRNB based on multiple arguments and consensus between physicians from complementary subspecialities (including radiologist, neurosurgeon, and radiation and medical oncologist), location within the irradiated volume, timing relative to radiotherapy, evolution of primary disease, and (in selected cases) biopsy and/or ^18^F-FDG PET/CT.

Data concerning patient characteristics, patient history and clinical status, diagnosis of fRNB, bevacizumab treatment disposition, adverse events (graded according to the Common Terminology Criteria for Adverse Events (CTCAE) v5.0), and response assessments was extracted from their medical records.

The study was conducted in accordance with the Declaration of Helsinki and approved by the Ethics Committee of UZ Brussel (EC-2022-164). All patients signed informed consent allowing the use of their data for the purpose of this analysis.

### 2.2. Statistical Analysis

In this study, descriptive statistics were used to summarize and present the results. Demographic information, including age, sex, and primary tumor, were collected and summarized using frequency tables and proportions. For continuous variables, the median and standard deviation were calculated. Categorical variables, such as adverse events, were analyzed using frequency and percent distributions. As this is a retrospective case series, no statistical significance can be reported.

### 2.3. Imaging

T1-weighted gadolinium and T2-weighted FLAIR (fluid-attenuated inversion recovery) MRI images from one month prior to the first dose and after the last dose were reviewed. To determine the effect of the treatment, volumetry was performed on these images using a semi-automated technique (Brainlab AG, Munich, Germany).

## 3. Results

### 3.1. Baseline Characteristics

Between March 2016 and July 2022, 13 patients (six females and seven males) with a median age of 52 years (range: 33–68 years) were treated with low-dose bevacizumab for fRNB. Baseline patient characteristics are summarized in Table 1. Among the patients, six patients (46%) had melanoma as a primary disease, three patients (23%) had non-small cell lung cancer, and one patient (8%) had an arteriovenous malformation. Seven patients (54%) received SRS, three patients (23%) received SRT, and two patients (23%) received both. Two out of the seven and one out of three patients who, respectively, received SRS and SRT had multiple courses for different lesions. One patient received two SRT courses for the same lesions. Four patients (31%) were initially treated with corticosteroids prior to receiving bevacizumab but had unsatisfactory outcomes. Three out of these four patients received concomitant corticosteroids at the initiation of BEV. The steroid dose was gradually reduced in these patients. Eight (62%) and four (31%) patients received, respectively, immunotherapy and chemotherapy between SRS/SRT and the first dose of bevacizumab. The median volume of FLAIR-hyperintensity at baseline was 20.1 cm^3^ (range: 2.74–98.3 cm^3^) and 4.49 cm^3^ for the T1 enhancing lesion (range: 1.79–21.3 cm^3^) on T1-weighted gadolinium MRI. The majority (69%) of the patients had a single fRNB lesion.

The diagnosis of fRNB was based on the medical history and MRI of the brain in all patients and was further complemented with ^18^F-FDG PET-CT in six patients and with a surgical biopsy in six patients. Median time between the first course of SRS/SRT and the start of the low-dose BEV regimen was 39 months (range: 18–90 months).

All patients had at least one fRNB-associated neurological symptom at the start of the treatment, including focal neurological deficits (e.g., paresis) (69%, *n* = 9), epileptic seizures (46%, *n* = 6), and headache (8%, *n* = 1,) (Table 1 and Table 2).

### 3.2. Treatment Disposition

**Figure 1:**
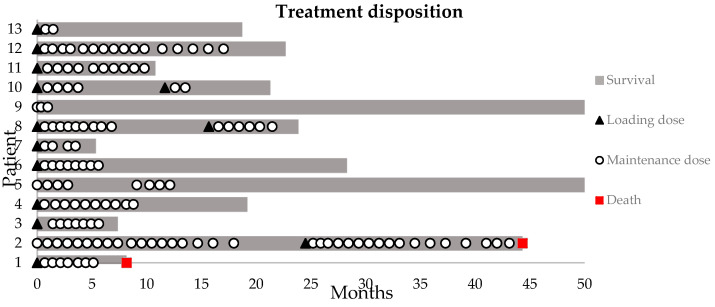
Swimmer plot of the bevacizumab disposition. Loading dose = 400 mg I.V.; maintenance dose = 100 mg I.V. Survival indicates the time between first loading dose and data base lock or death.

All patients, except three, received a loading dose of 400 mg bevacizumab I.V at the start of treatment. A median of seven maintenance doses (100 mg) was given (range: 2–38 doses). Two out of the three patients who did not receive a loading dose had previously received a conventional dose regimen of bevacizumab. Prolonging the interval between administrations from Q4w to Q6w in three patients after the initial response did not result in an increase in symptoms. In four patients, bevacizumab treatment was resumed following elective discontinuation. In one of these patients because of recurring symptoms, in two other patients following an asymptomatic increase in T1-weighted gadolinium and T2-weighted FLAIR MRI volume, and in one patient (25%, *n* = 4) because of both MRI changes, as well as an increase in symptoms (Figure 1). On average, patients were given 2–4 doses of BEV before their first evaluation. At this evaluation, treatment was either stopped or continued based on clinical symptoms and MRI response.

### 3.3. Clinical Outcome

Eleven patients (84.6%) experienced a complete symptomatic improvement during treatment. Six patients suffered from epileptic seizures within three months preceding the initiation of BEV; three of these patients suffered from therapy refractory epilepsy (defined by refractory to two or more anti-epileptic drugs). These patients experienced a marked improvement in refractory epileptic seizures, including two patients who became seizure-free. The other three patients experiencing epileptic seizures all became seizure-free while under the low-dose regimen. All patients continued their anti-epileptic medication. The WHO-PS (World Health Organization Performance Status) remained the same in nine patients (69%) while on treatment, improved in three patients (23%), and decreased in one patient (8%) (Table 1 and Table 2)

### 3.4. Objective Response on MRI of the Brain

All patients underwent at least one MRI scan at baseline and on treatment. Slice thickness ranged between 0.5 and 2 mm, depending on the MRI apparatus. T2-weighted FLAIR MRI-images demonstrated a decrease in the volume of edema in all patients. The median relative decrease in the FLAIR-hyperintensity volume was 81.1% (range: 7.1–95.4%). Three patients (23%) relapsed after electively discontinuing treatment following symptomatic and radiographic improvement while on treatment. After reinitiating treatment, these patients had a renewed radiographic decrease in the edema volume.

**Figure 2:**
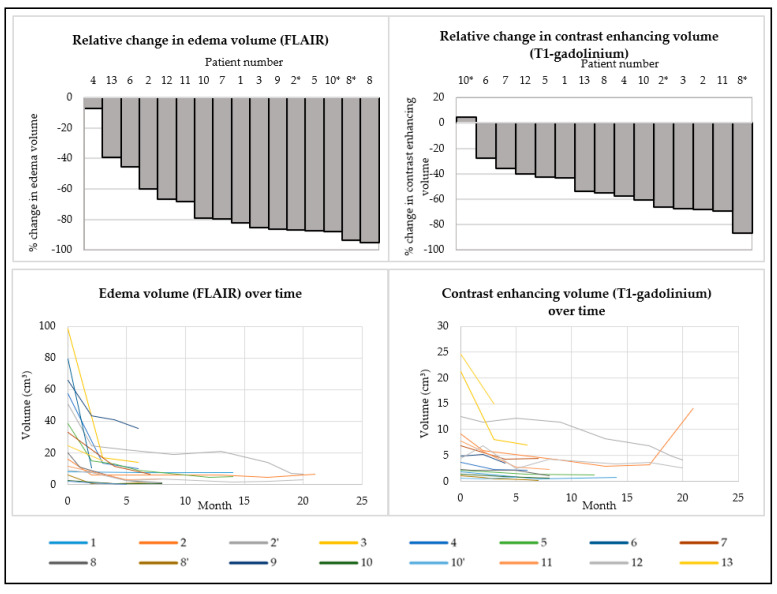
Change in T2-weighted FLAIR-hyperintensity and T1-weighted gadolinium enhancing volumes between baseline MRI and the most recent MRI at the end of BEV treatment (first row). Evolution in time of T2-weighted FLAIR-hyperintensity and T1-weighted gadolinium enhancing volumes during treatment (second row). * Indicates a re-challenge with a low-dose bevacizumab regimen after an elective discontinuation of bevacizumab treatment.

All patients who were evaluated by T1-weighted gadolinium MRI of the brain (*n* = 12) had a decrease in the contrast enhancing volume. One patient had a slight increase in the contrast enhancing volume after re-challenge, but a decrease in FLAIR-hyperintensity over the same period (Figure 2).

### 3.5. Safety

In general, the treatment was well-tolerated, and the adverse events were mild (Table 3). No unexpected adverse events were observed. There were no Grade 3 or higher adverse events. None of the patients needed to receive medical therapy for adverse events. One patient had to discontinue treatment because of a Grade 2 wound dehiscence (which was successfully treated with local treatment).

### 3.6. Survival

The median time between the first dose of bevacizumab and the database lock was 22 months (range: 5–56 months). Two patients died during this follow-up period due to progressive disease of their primary tumor (lung and melanoma). All the other patients remained alive at the time of the database lock and were still in follow up. Only one of patient remains in treatment.

## 4. Case Illustrations

### 4.1. Case Illustration 1

**Figure 3:**
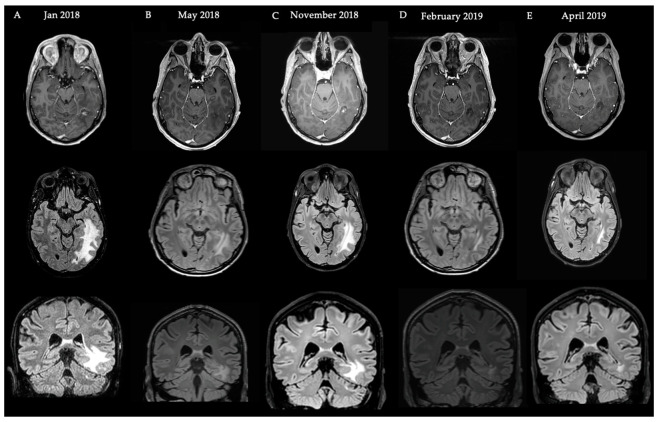
Schematic overview of events and MRI images with regard to the Case Illustration 1. First row: T1-weighted gadolinium MRI. Second row: T2-weighted FLAIR MRI (axial). Third row: T2-weighted FLAIR MRI (coronal) illustrating fRNB crossing over the tentorium.

We present the case of a 33-year-old male patient with a cerebellar medulloblastoma, who initially presented in 2011 with orthostatic headache. Surgical resection and radiotherapy were performed in 2011 (20 × 1.8 Gy full neuraxis + 10 × 1.8 Gy boost locally) and 2013 (recurrence, 26 × 2 Gy). In 2016, perilesional edema was visualized on follow-up MRI, and the patient experienced epileptic seizures. Therefore, a treatment of standard-dosing 7.5 mg/kg Q4w bevacizumab as well as levetiracetam was initiated. After four doses of bevacizumab, T2-weighted FLAIR MRI demonstrated a decrease in the perilesional edema and the patient did not experience epileptic seizures anymore, after which the treatment was discontinued. In 2018, he experienced a new epileptic seizure and T2-weighted FLAIR MRI demonstrated an increase in perilesional edema (A). Despite increasing the dosage of the anti-epileptic drug levetiracetam, the patient still experienced seizures. Therefore, a low-dose bevacizumab regimen of 100mg Q4w was initiated (omitting the 400mg loading dose). After four doses, treatment was discontinued because the patient did not experience epileptic seizures anymore and a decrease in volume on T2-weighted FLAIR MRI was observed (C). The patient remained in follow up every three months (clinical and MRI). After six months, a T2-weighted FLAIR MRI demonstrated a new increase in edema (D) prompting a restart of low-dose BEV. Another four doses later, he again had a reduction in edema volume, after which treatment was permanently discontinued (E). Two months after discontinuing the treatment, no increase in edema was visualized (F). The patient remained asymptomatic up to the last follow up (more than 2.5 years after the last dose of bevacizumab) (Figure 3).

### 4.2. Case Illustration 2

**Figure 4:**
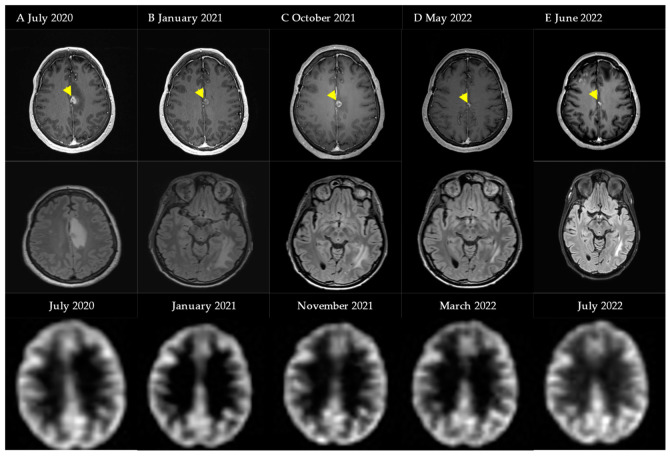
Schematic overview of events and MRI images with regard to Case Illustration 2. First row: T1-weighted gadolinium MRI (abnormality marked by arrow). Second row: T2-weighted FLAIR MRI. Third row: ^18^F-FDG PET-CT.

The second case is a 52-year-old female patient who presented with two melanoma brain metastases for which she received SRT in January and March of 2019. In July 2020 (A), a diagnosis of fRNB was made based on T1-weighted gadolinium and T2-weighted FLAIR MRI, further confirmed by a hypometabolic spot on ^18^F-FDG PET-CT. Because of frontal and parietal left-sided headaches, a low-dose regimen bevacizumab was initiated, starting with a loading dose of 400 mg, followed by a Q4w 100 mg I.V. maintenance dose. In January 2021, a decrease in edema on T2-weighted FLAIR MRI was observed (B). Treatment was interrupted because of diarrhea and minimal improvement in symptoms. Nine months later, because of an increase in edema (C) a new low-dose regimen was started with a 400 mg I.V. loading dose. In January 2022, she received SRS for a new metastatic lesion but continued BEV maintenance. After six maintenance doses, BEV was stopped because of radiological improvement (D). Edema on this MRI is a result of the intracranial progression of her melanoma for which new systemic treatment options were started. Six weeks after stopping BEV, no subsequent increase in edema was visualized (E) (Figure 4).

## 5. Discussion

Focal radiation necrosis of the brain (fRNB) is an important and increasingly common late adverse event of stereotactic radiotherapy and radiosurgery. Our case series confirms the observations in the literature, indicating that fRNB is more frequent after SRS, as compared to SRT (10 out of 13 patients received SRS). When symptoms occur, treatment is indicated. Depending on the localization and the prognosis of the patient, surgical debridement or systemic corticosteroids are currently considered as standard treatment options. Bevacizumab has been proven to be an effective alternative, avoiding the side effects resulting from prolonged administration of corticosteroids [29]. Gonzalez et al. reported that using a dosing regimen of 5 to 7.5 mg/kg bevacizumab administered every 2–3 weeks reduced fRNB-related neurocognitive deficits and cerebral edema [32]. Later, an observational study and a randomized controlled trial confirmed these finding [29,33]. However, until recently, bevacizumab has not been widely adapted as the preferred treatment option, as the high drug costs remain an important hurdle. More recently, bevacizumab biosimilars have been introduced, rendering the use of this drug more accessible. Our research group was among the first to report the effect of a fixed, low-dose bevacizumab regimen [35]. We have summarized all available evidence regarding the use of bevacizumab for the indication of fRNB in Table 4. Most data relate to retrospective case series, with important variations in sample size and patient heterogeneity.

This study included 13 patients who received at least one administration of 100 mg I.V. bevacizumab between 2016 and 2022. Ten of these patients received an initial loading dose of 400 mg, followed by a monthly maintenance dose of 100 mg. ^18^F-FDG PET-CT was useful to differentiate fRNB from brain metastasis, and therefore, can aid in diagnosis. This is in line with recent reviews, although further research is still needed [48,49]. While the study population is heterogeneous in their primary disease, all patients were treated for fRNB presenting after SRS/SRT. Within this population, 92% of the patient population experienced an improvement in clinical symptoms. Almost all the patients experiencing epileptic seizures became seizure-free under anti-epileptic medication in combination with low-dose bevacizumab, including two patients with refractory epileptic seizures. All patients had a radiographic decrease in edema volume on T2-weighted FLAIR MRI after starting BEV. These findings are in line with other reports on lower-dose regimens of bevacizumab for fRNB [36,37] (see Table 4 for a full overview). We first reported the results of a fixed-dose regimen, as opposed to weight-based dosing. The fixed doses used in this regimen were generally well-tolerated with only mild adverse events, and no new safety signs were observed. Almost all patients (10 out of 13) received an initial loading dose, followed by at least two maintenance doses (100 mg) before their first evaluation (=after 3 months) to decide on whether to continue treatment. This is in line with what is described in the literature (2–4 initial courses of BEV, both for a lower dose, as well as a conventional dose). In cases where a significant beneficial effect on MRI imaging is observed, as well as symptomatic improvements without a remaining need for corticosteroids, we will discuss the possibility of treatment interruption with the patient. Further dosing is adapted to patient symptoms and imaging.

Notwithstanding the limited number of patients in this retrospective single-center data collection, our preliminary data suggest that a low-dose regimen bevacizumab comprising a fixed loading dose of a 400 mg I.V. dose and a maintenance dose of 100 mg Q4w can be a simple and effective treatment option for symptomatic fRNB. While the design comes with its inherent risk of biases, we think the real-world experience described in this manuscript demonstrates a uniform and clinically meaningful activity in all patients treated with this treatment regimen. In the future, more data on outcome according to primary pathology should be collected within the context of a prospective clinical trial. Based on our results, this regimen is a valid cost-sparing alternative for the expensive standard dose of 5 to 7.5 mg/kg of BEV every 3–4 weeks, especially since the recent availability of bevacizumab biosimilars and the subsequent reduced drug cost. We have, therefore, adapted this regimen as our institutional standard of care treatment regimen for fRNB that is not amenable for neurosurgical resection.

## 6. Conclusions

Our preliminary data demonstrate that treatment of fRNB with a low-dose regimen of bevacizumab can be an effective and cost-lowering alternative for standard-dose bevacizumab and likely has fewer side effects as compared to long-term high-dose corticosteroid use. ^18^F-FDG PET/CT can be a useful supplementary imaging modality to differentiate fRNB from malignant brain lesion recurrence or metastasis. Further research is needed to prospectively validate this low-dose treatment protocol in larger studies and homogenous patient cohorts.

## Figures and Tables

**Table 1:** Baseline patient characteristics. fRNB = focal radiation necrosis of the brain, WHO-PS = World Health Organization Performance Status.

Characteristics	No. of Patients (%) Total (*n* = 13)
Median age, years (range)	52 (33–68)
Sex	
Male	7 (54%)
Female	6 (46%)
Primary disease	
Melanoma	6 (46%)
Non-small cell lung carcinoma	3 (23%)
Breast cancer	1 (8%)
Renal cell carcinoma	1 (8%)
Medulloblastoma	1 (8%)
Arteriovenous malformation	1 (8%)
WHO-PS at inclusion	
0	10 (77%)
1	3 (23%)
Number of fRNB locations	
1	9 (69%)
2	3 (23%)
3	1 (8%)
Prior radiation therapy	
Stereotactic radiosurgery	6 (46%)
Stereotactic radiotherapy	3 (23%)
Both	4 (31%)
Corticosteroid treatment prior to initiation of bevacizumab	
Yes	5 (39%)
No	8 (62%)
Diagnosis of fRNB based on MRI supported by	
Histopathological examination of biopsy	6 (46%)
^18^F-FDG PET-CT	6 (46%)
MRI-only	3 (23%)
Symptoms at baseline	
Headache	1 (8%)
Epileptic seizures	6 (46%)
Focal neurological deficit	9 (69%)

**Table 2:** Individual patient characteristics. BEV = bevacizumab, AVM = arteriovenous malformation, BC = breast cancer, MB = medulloblastoma, MEL = melanoma, NSCLC = non-small cell lung carcinoma, RCC = renal cell carcinoma, IPI = ipilimumab, NIVO = nivolumab, PEMBRO = pembrolizumab, Y = yes, N = no, Y/N = mixed, ICI = immune checkpoint inhibitor. ^1^ indicates concomitant continuation of corticosteroids. Time expressed in months.

Patient	Primary Disease	Location of fRNB	Radiation Therapy	Prior ICI	Prior Chemotherapy	Time since SRS/SRT	WHO-PS Evolution	Time on Treatment	Re-challenge Needed	Steroids Prior	Symptomatic Improvement
**1**	NSCLC	Right frontal	SRS (1 × 20 Gy)			23	1 -> 1	5	N	Y^1^	Y
**2**	MEL	Left parietal	SRS (1 × 20 Gy)	IPI + NIVO + PEMBRO		43	0 -> 1	40	Y	N	Y/N
**3**	NSCLC	Bilateral parietal	SRS (20 Gy)	PEMBRO		37	0 -> 1	5	N	N	Y
**4**	RCC	Right frontoparietal	SRS (20 Gy) + SRT (5 × 7.5 Gy)	NIVO		58	0 -> 0	8	N	N	Y
**5**	MB	Left supratentorial	SRT (doses see case report 1)		Carboplatin + etoposide	79	0 -> 0	11	Y	N	Y
**6**	AVM	Right parietal	SRS (1 × 20 Gy)			79	0 -> 0	5	N	N	Y
**7**	BC	Left temporal	SRS (1 × 20 Gy) + SRT (5 × 7 Gy)		Paclitaxel	90	0 -> 0	3	Y	N	Y
**8**	MEL	Left frontoparietal	SRT+SRS (see case report 2)	IPI + NIVO		18	1 -> 1	11	Y	Y^1^	Y
**9**	MEL	Right frontal	SRS (1 × 20 Gy)	IPI + NIVO + PEMBRO		60	0 -> 1	1	N	Y^1^	Y
**10**	MEL	Bilateral frontoparietal	SRS (1 × 20 Gy)	IPI + NIVO	Temozolomide	38	0 -> 0	4	Y	N	Y
**11**	MEL	Left cerebellar	SRT (3 × 9 Gy)			24	0 -> 0	10	N	N	Y
**12**	MEL	Right cerebellum	SRT (4 × 8 Gy)	PEMBRO		39	0 -> 0	20	N	N	Y
**13**	NSCLC	Right temporal	SRS (1 × 20 Gy) + SRT (5 × 7 Gy)	PEMBRO	Cisplatin + docetaxel	32	1 -> 0	2	N	Y	N

**Table 3:** Adverse events according to the Common Terminology Criteria for Adverse Events (CTCAE) v5.0.

All AE	*n* = 6			
	Grade I	Grade II	Grade III	Grade IV
Arterial Hypertension (*n* [%])	2 (15%)	0	0	0
Headache (*n* [%])	1 (8%)	1 (8%)	0	0
Alopecia (*n* [%])	2 (15%)	0	0	0
Mucositis oral (*n* [%])	2 (15%)	0	0	0
Diarrhea (*n* [%])	1 (8%)	0	0	0
Epistaxis (*n* [%])	1 (8%)	0	0	0
Proteinuria (*n* [%])	0	1 (8%)	0	0
Wound dehiscence (*n* [%])	0	1 (8%)	0	0

**Table 4:** Overview of available evidence regarding the use of bevacizumab for the treatment of focal radiation necrosis of the brain.

Citation	Study Design	No. of Patients	BEV Dose Regimen	Primary Pathologies (*n*)	Radiation (*n*)	Radiographic Response Rate (%)	Symptomatic Improvement (%)	Adverse Events Described (*n*)
Gonzalez et al., 2007. USA. [32]	Retrospective	8	5 mg/kg q2w or 7.5 mg/kg q3w	AOA (1), AOD (1), AA (1), HP (1), GBM (4)	RT/SRS	100%		None
Tye et al., 2014. USA. [29]	Review	71	7.5 mg/kg q2w	GBM (22), Met (11), pG (15), Men (6), NPC (5), other (5), ponG (3), HP (2), AVM (2)	EBRT (57), SRS (9), BNCT (3), PT (2)	100%		Small vessel thrombosis (3), sagittal sinus thrombosis (1), aspiration pneumonia (1), pneumonia with sepsis (1), pulmonary embolus (1)
Levin et al., 2012. USA. [33]	Randomized controlled trial	7	7.5 mg q3w	NPC (2), MS (1), A (1), ODG (1), SCC (1), PA (1)	RT (7)	100%	100%	Small vessel thrombosis (3), aspiration pneumonia (1), pulmonary embolus secondary from a deep vein thrombosis (1), superior sagittal sinus thrombosis (1)
Wang et al., 2012. China. [38]	Retrospective	17	7.5 mg/kg q2w	GBM (7), Met (6), AA (1), AVM (1), Men (1), ODG (1)	EBRT (12), SRS (4), FSRT (2)	100%	94.1%	Hypertension (1), proteinuria (1), temporary fatigue (1)
Boothe et al., 2013. USA [39].	Retrospective	11	7.5 mg/kg q2w or 10 mg/kg q3w or 15 mg/kg q6w	Met (11)	SRS (11), WBRT (5)	100%	63.6%	None
Furuse et al., 2013. Japan. [40]	Retrospective	11	5 mg/kg q2w	GBM (4), AM (3), Met (3), AA (1)	XRT (7), SRS (6), BNCT (3), SRT (1),	100%		None
Yonezawa et al., 2014. Japan. [41]	Non-randomized clinical trial	9	5 mg/kg q2w	GBM (6), Met (2), ODG (1)	GTV (6), SRT(2), EL (1), SRT (1), WBRT (1) PT (1)	100%		Anemia, thrombocytopenia, lymphocytopenia, and/or neutropenia (3)
Sadraei et al., 2015. USA. [42]	Retrospective	24	5 mg/kg q2w or 7.5 mg/kg q3w or 10 mg/kg q2w or 15 mg/kg q3w	Met (17), GBM (2), AVM (2), ODG (1), AE (1), TA (1)	WBRT (10), SRS (18), PT (1)	95.8%	95.8%	Grade 2 or less: hypertension, fatigue, urinary tract infection, and proteinuria (6). Grade 3: pulmonary embolism (1)
Zhuang et al. China. 2016. [36]	Non-randomized clinical trial	21	1 mg/kg q3w	Met (21)	SRS (16), WBRT (5)	95.2%	81%	Allergy (1), hypertension (1)
Zhuang et al. China. 2016. [43]	Retrospective	14	5 mg/kg q3-4w	Met (14)	STI (10), WBRT (4)	92.9%	83.3%	Allergy (1), hypertension (1)
Weng et al., 2021. China. [37]	Retrospective	22	3 mg/kg q2w	Met (22)	SRS (22)	100%		None
Li et al., 2017. China. [44]	Retrospective	50	5mg/kg q2w	NPC	RT	76%		Not collected
Xu et al., 2018. China. [45]	Randomized, open label clinical trial	58	5 mg/kg q2w	NPC	RT	51.8%	62.1%	Hypertension (12), fatigue (7), infection (4), hemorrhage (4), insomnia (3), headache (3), rash (3), fever (2), blurred vision (1), hyperglycemia (1), stroke (1)
Alessandretti et al., 2021. Brazil. [46]	Retrospective	2	5 mg/kg q2w	Met (2)	SRS (2)	100%	50%	None
Glitza et al., 2017 USA. [47]	Retrospective	7	5, 7.5, 10 mg/kg	Melanoma	SRS/WBRT	57.4%	71.5%	Arthralgia (1), dysgeusia (1)
This study	Retrospective	13	400 mg loading dose, 100 mg q4w maintenance dose	Met (11), MB (1), AVM (1)	SRS (8), SRT (5)	100%	11	Hypertension (2), headache (2), mucositis oral (2), alopecia (2), diarrhea (1), epistaxis (1), proteinuria (1), wound dehiscence (1)

Abbreviations: AA = anaplastic astrocytoma, AOA = anaplastic oligoastrocytoma, AOD = anaplastic oligodendroglioma, AM = anaplastic meningioma, AVM = arteriovenous malformation, BEV = bevacizumab, BNCT = boron neutron capture therapy, EBRT = external beam radiation therapy, EL = extended local, FSRT = fractionated stereotactic radiotherapy, GBM = glioblastoma, GTV = gross tumor volume, HP = hemangiomapericytoma, MB = medulloblastoma, Men = meningioma, Met = metastasis from solid tumor, *n* = number of patients, NPC = nasopharyngeal carcinoma, ODG = oligodendroglioma, pG = primary glioma, ponG = pontine glioma, PT = proton therapy, RT = radiotherapy, SRS = stereotactic radiosurgery, SRT = stereotactic radiotherapy, STI = stereotactic irradiation, WBRT = whole brain radiotherapy, XRT = X-ray radiotherapy.

## Data Availability

Data are available on reasonable request to the corresponding author.
